# Dataset on cocoa farmers’ agrochemical handling practices and safety compliance in Ahafo Ano North district, Ashanti region, Ghana

**DOI:** 10.1016/j.dib.2019.104767

**Published:** 2019-11-06

**Authors:** Abayomi Samuel Oyekale

**Affiliations:** Department of Agricultural Economics and Extension, North-West University Mafikeng Campus, Mmabatho, 2735, South Africa

**Keywords:** Cocoa, Agrochemicals, Safety compliance, Occupational safety, Ghana

## Abstract

Agrochemicals are essential but hazardous inputs being utilized at different stages in cocoa production. Safeguarding the health of workers handling these chemicals is therefore of utmost importance. Although Ghanaian government implemented mass spraying of cocoa with every essential occupational safety being followed, non-workability of the programme in many parts of the cocoa producing areas necessitates supplementary application of agrochemicals by many farmers. Therefore, a survey was conducted in Ahafo Ano North district of the Ashanti region in 2015 to understand the compliance of farmers to safety guidelines in handling agrochemicals. The survey was conducted with structured questionnaires that were written in English language and translated into the local language in the course of the interviews. A total of 246 cocoa farmers were interviewed using stratified sampling procedures. The questionnaire, which was divided into four sections solicited information on farmers’ socioeconomic characteristics, safeguard measures being taken by the farmers in the course of handling agrochemicals, health complaints after handling agrochemicals and stress and occupational hazards. The dataset is herewith made available and it is considered of vital usefulness given some serious policy implications of occupational health hazards among cocoa farmers.

**Table:** Specifications Table

Subject	Agricultural Sciences
Specific subject area	Occupational Safety in Cocoa Production
Type of data	Table, charts and SPSS data file.
How data were acquired	Farming households' survey with structured questionnaire.
Data format	Raw and Analysed
Experimental factor	Face to face interviews were conducted with well structured questionnaires.
Experimental feature	Compliance with some standard occupational safety guidelines in handling agrochemicals.
Data source location	Ahafo Ano North District, Ghana
Data accessibility	Dataset is hereby submitted with the article.
Related research article	Oyekale A.S. (2017). Non-Compliance with Agrochemical Safety Guides and Associated Health Risks among Cocoa Farmers in the Ashanti Region of Ghana. *African Journal of Biomedical Research*, 20 (1), 9–15. https://www.ajol.info/index.php/ajbr/article/view/155639/145268

**Table:** 

**Value of the Data** The dataset is valuable for the following reasons: • The data are useful because they provide information on compliance of cocoa farmers to standard occupational safety guidelines while handling agrochemicals; • The data provides some insights for researchers and key stakeholders in the cocoa industry on the safety knowledge and behaviours of cocoa farmers in handling agrochemicals. • These data can be further analysed to understand the correlates of farmers' compliance with agrochemical safety guidelines in respect of wearing of personal protective equipment, • The data can also promote our understanding of the factors explaining use and re-use of agrochemical containers and effective disposal of other agrochemical containers; • The data also provide information on the basic health complaints from farmers after handling agrochemicals and their choices of healthcare services for treatments.

## 1 Data

In this paper, a dataset that was collected from 246 cocoa farmers in June 2015 was presented. The aim of the survey was to understand some safety precautions being taken by cocoa farmers in the course of handling agrochemicals and associated health complaints. The dataset provides researchers with some variables that can be used to explore research topics on issues of occupational hazards from mishandling of agrochemicals and health complaints among cocoa farmers. The subject of agrochemical usage in agricultural production is vital and for cocoa, it is of critical relevance given the spectrum of pests and diseases being associated with cocoa plant [[1], [2], [3]]. The dataset attempt to profile some socioeconomic characteristics of the farmers. Table 1 shows that majority of the farmers were males, married, attained primary education and primarily involved in farming occupation. The dataset contains other demographic variables that can be utilized to understand the differences in the behaviours of cocoa farmers on issues of compliance with agrochemical safety guidelines.

**Table 1:** Selected demographic characteristics of the respondents.

	Frequency	Percent	Cumulative Percent
Single	15	6.1	6.1
Married	188	76.4	82.5
Divorced	13	5.3	87.8
Widowed	30	12.2	100.0
*Gender*			
Male	150	61.0	61.0
Female	96	39.0	100.0
*Educational Level*			
Primary education	117	47.6	47.6
Secondary education	41	16.7	64.2
Tertiary education	3	1.2	65.4
None	85	34.6	100.0
*Primary Occupation*			
Farming	227	92.3	92.3
Artisan	6	2.4	94.7
Trading	8	3.3	98.0
Civil service	5	2.0	100.0

**Fig. 1:**
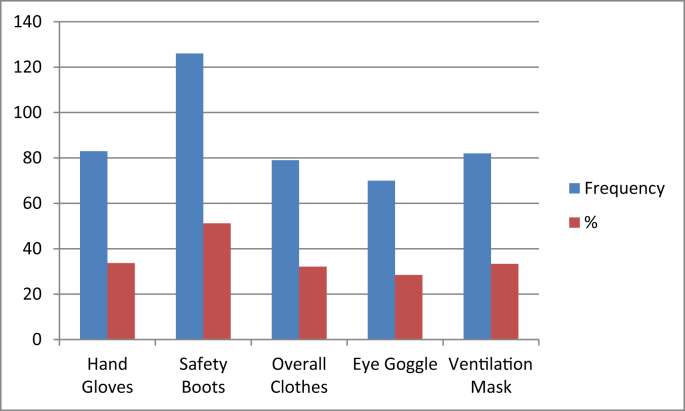
Ownership of Personal Protective Equipment (PPE) by cocoa farmers.

The data also contain information on the types of agrochemicals that were being used by cocoa farmers, awareness of precautionary measures to be taken in the course of handling agrochemicals, ownership and use of basic safety kits (hand gloves, safety boot, overall protective clothe, eye protective goggle and ventilation mask) while handling agrochemicals and understanding of the right way of agrochemical application and disposal of containers/leftovers. Fig. 1 shows that safety boots recorded the highest ownership with 51.22%. This is followed by hand gloves (33.74%), ventilation masks (33.33% and overall cloth (32.11%). The data also contain information on contacts with agrochemicals, post-handling health complaints, past emergency cases from inadequate handling of agrochemicals and stress and occupational hazard exposures.

## 2 Experimental design, materials and methods

The survey that resulted into generation of this dataset was conducted in June 2015 at Ahafo Ano North district in Ashanti region of Ghana. The district was purposefully chosen because it is among the top cocoa growing areas in the region. With the assistance of residence extension officers, we employed stratified random sampling procedure with sample size selected in proportion to the estimated number of cocoa farmers in each stratum. The district was stratified into twenty main communities based on prominence of farming. Out of these twenty strata, eight were randomly selected and sample sizes were allocated based on estimated number of cocoa farmers as provided by the extension officers. The sampled communities were Akwasiase (125), Bonkrom (42), Tepa (37), Abonsuaso (14), Jacobu (9), Kwekwewere (9), Dwahoo (6) and Anyinasuso (4). The enumerators were largely farm extension officers who were working directly with cocoa farmers in the district. Prior to the commencement of the survey, the enumerators were properly trained on the requirements of the survey and a pre-testing of the questionnaire was undertaken among few farmers cocoa farmers. In each of the selected communities, the leaders of the cocoa farmers’ groups and/or the chiefs assisted the extension agents in informing cocoa farmers on the purpose of the survey. Although the questionnaire (which is also made available with the dataset) was designed in English language, interviews were conducted for majority of the farmers in their local language (Akan-twi).

## Funding

The funding for this dataset was obtained from the 2014 IREA of the researcher as approved by the North-West University Management Council.
