# Supplementary material for: Dataset on cocoa farmers’ agrochemical handling practices and safety compliance in Ahafo Ano North district, Ashanti region, Ghana
Source: Data Brief. 2019 Nov 6;27:104767. doi: 10.1016/j.dib.2019.104767 (PMC6864171; doi:10.1016/j.dib.2019.104767)
Supplement: Multimedia component 1 [file mmc1.docx]

**AGROCHEMICAL SAFEGUARD MEASURES AND HEALTH OF COCOA FARMERS**

Dear Sir/Ma,

This questionnaire is designed to obtain information on above project topic. We are soliciting for your assistance in providing the requested information as outlined in the questionnaire. All information provided by you shall be treated as confidential and used strictly for research purpose. Thank you very much.

**SECTION A: SOCIO-ECONOMIC CHARACTERISTICS OF COCOA FARMERS**

1. Name of village……………………………………………… ii. District………………………………………
2. Age of the farmers ………………………………………………. (years)
3. Age of the household heads ……………………………………………. (years)
4. Marital status: (a) single [ ] (b) married [ ] (c) divorced [ ] (d) widowed [ ]
5. Gender: (a) male[ ] (b) female [ ]
6. Level of education: (a) primary education [ ] (b) secondary education [ ] (c) tertiary education [ ]

(d) adult education [ ] (e) none[ ]

1. What is your primary occupation? (a) Farming [ ] (b) artisan [ ] (c) trade [ ] (d) civil servant [ ]

(e) transport [ ] (f) others specify………………………..

1. What is your secondary occupation? (a) Farming [ ] (b) artisan [ ] (c) trade [ ] (d) civil servant [ ]

(e) transport [ ] (f) others specify………………………..

1. What is your household size? ..........................................................
2. How many years of formal schooling did you have? ………………………………………
3. For how many years have you been into farming? ……………………………………………
4. How many years have you been into cocoa farming? …………………………………………

(13). What is the total area of land cultivated to cocoa on your farm? …………………………….

(13b) How do rate your health? i. Excellent …. ii Very Good …. iii. Good …. iv. Bad …. v. Worse ……..

**SECTION B: SAFEGUARD MEASURES**

14. Do you use agrochemicals on your cocoa farms farm? (a) yes [ ] (b) no[ ]

15. If yes, how often do you use it……………………………………………………………

16. What type of agrochemicals do you use on your cocoa farms?

(Please kindly tick the ones you normally use and indicate the trends of usage in the past four years).

| Agrochemicals | Liquid | Solid | Trend |
| --- | --- | --- | --- |
| Pesticides | Yes….No… | Yes No… | Increasing…. Decreasing … Same … |
| Insecticides | Yes….No… | Yes No… | Increasing…. Decreasing … Same … |
| Herbicides | Yes….No… | Yes No… | Increasing…. Decreasing … Same … |
| Fertilizer | Yes….No… | Yes No… | Increasing…. Decreasing … Same … |
| Fungicide | Yes….No… | Yes No… | Increasing…. Decreasing … Same … |

17. Are you aware that there are some safety measures to be taken while applying these chemicals?

| Agrochemicals | Response |
| --- | --- |
| Pesticides | Yes…. No… |
| Insecticides | Yes…. No… |
| Herbicides | Yes…. No… |
| Fertilizer | Yes…. No… |
| Fungicide | Yes…. No… |

18. If yes, what are the precautions you normally take?

| Precautions | Response |
| --- | --- |
| Do not handle with bare hand | Yes…. No… |
| Do not keep within the reach of children | Yes…. No… |
| Do not inhale the chemical | Yes…. No… |
| Do not pour the left over inside river or stream | Yes…. No… |
| Have a pit dug within the farm where left over of chemicals are poured | Yes…. No… |
| Ensure no contact of chemicals with food or drink | Yes…. No… |
| Ensure it is kept under specified temperature | Yes…. No… |

19. Do you have any of the following kits and how much did you procure them?

| Safeguard kits | Have it | Price |
| --- | --- | --- |
| Hand gloves | Yes…. No… |  |
| Safety boots | Yes…. No… |  |
| Overall | Yes…. No… |  |
| Goggle | Yes…. No… |  |
| Ventilation mask | Yes…. No… |  |

20. Do you follow the manufacturer’s instruction before using the following agrochemicals?

| Agrochemicals | Response |
| --- | --- |
| Pesticides | Yes…. No… |
| Insecticides | Yes…. No… |
| Herbicides | Yes…. No… |
| Fertilizer | Yes…. No… |
| Fungicide | Yes…. No… |

21. When you spray the following chemicals, how do you carry them?

| Agrochemicals | Back it | Carry on my chest | Lift with hands from place to place | Carry on my shoulder |
| --- | --- | --- | --- | --- |
| Pesticides | Yes…. No… | Yes…. No… | Yes…. No… | Yes…. No… |
| Insecticides | Yes…. No… | Yes…. No… | Yes…. No… | Yes…. No… |
| Herbicides | Yes…. No… | Yes…. No… | Yes…. No… | Yes…. No… |
| Fertilizer | Yes…. No… | Yes…. No… | Yes…. No… | Yes…. No… |
| Fungicide | Yes…. No… | Yes…. No… | Yes…. No… | Yes…. No… |

23. If you must carry chemicals on your back during spraying, do you have someone to assist you in lifting it up until it is properly fixed at your back?

| Agrochemicals | Response |
| --- | --- |
| Pesticides | Yes…. No… |
| Insecticides | Yes…. No… |
| Herbicides | Yes…. No… |
| Fertilizer | Yes…. No… |
| Fungicide | Yes…. No… |

24. When spraying the following agrochemicals on cocoa farm, do you spray along the wind direction?

| Agrochemicals | Response |
| --- | --- |
| Pesticides | Yes…. No… I don’t know ……….. |
| Insecticides | Yes…. No… I don’t know ……….. |
| Herbicides | Yes…. No… I don’t know ……….. |
| Fertilizer | Yes…. No… I don’t know ……….. |
| Fungicide | Yes…. No… I don’t know ……….. |

25. Do you eat or drink during the process of spraying the following agrochemicals without having removed the safeguard kits and washing your hands properly?

| Agrochemicals | Response |
| --- | --- |
| Pesticides | Yes…. No… |
| Insecticides | Yes…. No… |
| Herbicides | Yes…. No… |
| Fertilizer | Yes…. No… |
| Fungicide | Yes…. No… |

26. When using the following agrochemicals, do you always wear the following kits?

| Agrochemicals | Hand gloves | Safety boots | Overall | Goggle | Ventilation mask |
| --- | --- | --- | --- | --- | --- |
| Pesticides | Yes…. No… | Yes…. No… | Yes…. No… | Yes…. No… | Yes…. No… |
| Insecticides | Yes…. No… | Yes…. No… | Yes…. No… | Yes…. No… | Yes…. No… |
| Herbicides | Yes…. No… | Yes…. No… | Yes…. No… | Yes…. No… | Yes…. No… |
| Fertilizer | Yes…. No… | Yes…. No… | Yes…. No… | Yes…. No… | Yes…. No… |
| Fungicide | Yes…. No… | Yes…. No… | Yes…. No… | Yes…. No… | Yes…. No… |

27. After applying the agrochemicals, what do you do to the chemical containers? (a) wash and use at home[ ] (b) wash and use on the farm [ ] (c) properly bury it [ ] (d) throw it anywhere on the farm [ ]

28. After applying the agrochemicals, what do you do to the left over of the chemicals?

| Agrochemicals | Retain for use next time | Pour inside running river/stream | Pour anywhere on the cocoa farm | Pour on nearby cocoa trees | Pour inside a hole dug for that purpose | Pour inside a special drum on the farm. |
| --- | --- | --- | --- | --- | --- | --- |
| Pesticides | Yes…. No… | Yes…. No… | Yes…. No… | Yes…. No… | Yes…. No… | Yes…. No… |
| Insecticides | Yes…. No… | Yes…. No… | Yes…. No… | Yes…. No… | Yes…. No… | Yes…. No… |
| Herbicides | Yes…. No… | Yes…. No… | Yes…. No… | Yes…. No… | Yes…. No… | Yes…. No… |
| Fertilizer | Yes…. No… | Yes…. No… | Yes…. No… | Yes…. No… | Yes…. No… | Yes…. No… |
| Fungicide | Yes…. No… | Yes…. No… | Yes…. No… | Yes…. No… | Yes…. No… | Yes…. No… |

**SECTION C: SAFEGUARD MEASURES AND HEALTH**

29. Does any of the following happen when using the following agrochemicals?

| Agrochemicals | Inhale it | Taste it | Drink it | Touch with bare hand | Enter my eyes | Touch my skin |
| --- | --- | --- | --- | --- | --- | --- |
| Pesticides | Yes…. No… | Yes…. No… | Yes…. No… | Yes…. No… | Yes…. No… | Yes…. No… |
| Insecticides | Yes…. No… | Yes…. No… | Yes…. No… | Yes…. No… | Yes…. No… | Yes…. No… |
| Herbicides | Yes…. No… | Yes…. No… | Yes…. No… | Yes…. No… | Yes…. No… | Yes…. No… |
| Fertilizer | Yes…. No… | Yes…. No… | Yes…. No… | Yes…. No… | Yes…. No… | Yes…. No… |
| Fungicide | Yes…. No… | Yes…. No… | Yes…. No… | Yes…. No… | Yes…. No… | Yes…. No… |

30. After spraying agrochemicals, do you suffer from any of the following health symptoms?

| Agrochemicals | Eye irritations | Skin irritations | Nasal discharge/ sneezing | Headache | Coughing | Breathing difficulties |
| --- | --- | --- | --- | --- | --- | --- |
| Pesticides | Yes…. No… | Yes…. No… | Yes…. No… | Yes…. No… | Yes…. No… | Yes…. No… |
| Insecticides | Yes…. No… | Yes…. No… | Yes…. No… | Yes…. No… | Yes…. No… | Yes…. No… |
| Herbicides | Yes…. No… | Yes…. No… | Yes…. No… | Yes…. No… | Yes…. No… | Yes…. No… |
| Fertilizer | Yes…. No… | Yes…. No… | Yes…. No… | Yes…. No… | Yes…. No… | Yes…. No… |
| Fungicide | Yes…. No… | Yes…. No… | Yes…. No… | Yes…. No… | Yes…. No… | Yes…. No… |

31. After using the following agrochemicals, do you suffer from any of the following pains?

| Agrochemicals | Lower abdominal pains | Upper abdominal pains | Neck pains | Backache | Waist pains | General body pains |
| --- | --- | --- | --- | --- | --- | --- |
| Pesticides | Yes…. No… | Yes…. No… | Yes. No… | Yes…. No… | Yes…. No… | Yes…. No… |
| Insecticides | Yes…. No… | Yes…. No… | Yes…. No… | Yes…. No… | Yes…. No… | Yes…. No… |
| Herbicides | Yes…. No… | Yes…. No… | Yes…. No… | Yes…. No… | Yes…. No… | Yes…. No… |
| Fertilizer | Yes…. No… | Yes…. No… | Yes…. No… | Yes…. No… | Yes…. No… | Yes…. No… |
| Fungicide | Yes…. No… | Yes…. No… | Yes…. No… | Yes…. No… | Yes…. No… | Yes…. No… |

32. In what way do you treat yourself when you suffer from the following as a result of spraying agrochemicals?

| Symptoms | How did you treat it when affected |
| --- | --- |
| Eye irritation | Public hospital … Private hospital … Self medication.. Chemist Traditional ….. Others …… |
| Skin irritation | Public hospital … Private hospital … Self medication.. Chemist Traditional ….. Others …… |
| Nasal discharge/sneezing | Public hospital … Private hospital … Self medication.. Chemist Traditional ….. Others …… |
| Headache | Public hospital … Private hospital … Self medication.. Chemist Traditional ….. Others …… |
| Coughing | Public hospital … Private hospital … Self medication.. Chemist Traditional ….. Others …… |
| General weakness | Public hospital … Private hospital … Self medication.. Chemist Traditional ….. Others …… |
| Upper abdominal pains | Public hospital … Private hospital … Self medication.. Chemist Traditional ….. Others …… |
| Lower abdominal pains | Public hospital … Private hospital … Self medication.. Chemist Traditional ….. Others …… |
| Neck pains | Public hospital … Private hospital … Self medication.. Chemist Traditional ….. Others …… |
| Backaches | Public hospital … Private hospital … Self medication.. Chemist Traditional ….. Others …… |
| Waist pains | Public hospital … Private hospital … Self medication.. Chemist Traditional ….. Others …… |
| General body pains | Public hospital … Private hospital … Self medication.. Chemist Traditional ….. Others …… |

33. Have you previously had any emergency situation arising from household members drinking agrochemicals?

| Agrochemicals | Mistakenly | Suicide attempt |
| --- | --- | --- |
| Pesticides | Yes…. No… | Yes…. No… |
| Insecticides | Yes…. No… | Yes…. No… |
| Herbicides | Yes…. No… | Yes…. No… |
| Fertilizer | Yes…. No… | Yes…. No… |
| Fungicide | Yes…. No… | Yes…. No… |

34. What are the coping strategies adopted in order to reduce the effect of the agrochemical on your health?

a. Drinking a tin of milk… b. Drinking of water. c. Bath immediately and apply body cream.

**SECTION D. STRESS AND OCCUPATIONAL HAZARD MANAGEMENT ON COCOA FARMS**

35. How often do you go through stress? i. Never ………….. ii. Sometimes iii. Often ……….

36. What often brings you into stressful conditions? (tick many) i. Finance… ii. Health problem .....

iii. Cocoa spraying……. iv. Marital challenges … v, Other farming …………….. vi. Clearing of cocoa farm……. vii. Black pod diseases.

37. Are you going through more stress from any of the following?

| Stressors | Yes/No | Stressors | Yes/No |
| --- | --- | --- | --- |
| Difficulties in getting drinking water |  | Reduced farm produce |  |
| Difficulties in getting cooking water |  | Loss of job |  |
| Difficulties in getting fuel wood |  | Divorcement |  |
| More crop pests |  | Lower wages |  |
| Difficulties in weed control |  | Sickness of household head |  |
| Difficulties in controlling crop diseases |  | Resentment at home |  |
| Sickness of household members |  | Difficulties in marketing cocoa beans |  |
| Long distance work to the farm |  | Long distance carrying of loads from farms |  |
| Low price of agricultural outputs |  | High price of farm inputs |  |
| Disease outbreak on cocoa farms |  | Lack of labour to use on cocoa farms |  |
| The danger of dwelling collapsing |  | Reduction in cocoa production |  |
| Ageing cocoa trees |  | Not having enough money |  |
| Not having enough food |  | Always falling sick |  |

38. What health problem do you suffer as a result of stress? i. Headache ……….. ii. Migraine ………

iii. Body pains …….. iv. Sleeplessness …….. v. Body weakness ……… vi. Others ………………

39. What do you do to ease your stress? i. To go church…………. ii. Go to mosque ……………….

iii. Drink alcohol ……………… iv. Go to party………………v. Hang out with friends………..

40. Is there any health problem you suffer from due to stress? i. Yes ……… No ……………………

41. Has it been confirmed by doctors that you suffer from high blood pressure? i. Yes …... No ………

42. Do you know this problem is associated with stress? i. Yes ……… No ……………………

43. Are you presently on daily drug in order to keep your blood pressure at the normal range?

i. Yes ……… No ……………………

44. Do you regularly check your blood pressure in hospital i. Yes ……… No ……………………

45. Please, provide information on smoking of cigarette and others stuffs among household members:

| Household head smokes | Yes/No | Neighbour smoke | Yes/No |
| --- | --- | --- | --- |
| Wife/wives smoke | Yes/No | Inhale cigarette smoke elsewhere | Yes/No |
| Other household member smoke | Yes/No |  |  |

46. What is the frequency of smoking or inhaling cigarette smokes among household members?

| Household head | Daily… Weekly… Monthly… | Neighbour smoke | Daily… Weekly… Monthly… |
| --- | --- | --- | --- |
| Wife/wives | Daily… Weekly… Monthly… | Inhale smoke | Daily… Weekly… Monthly… |
| Other household member | Daily… Weekly… Monthly… |  |  |

47. Please, provide average number of cigarette sticks being smoked by household members:

| Person | No. of cigarette sticks | Trend compared to last month |
| --- | --- | --- |
| Household head |  | Increasing,,….. Decreasing……. Same……. |
| Wife/wives |  | Increasing,,….. Decreasing……. Same……. |
| Other household member |  | Increasing,,….. Decreasing……. Same……. |

48. Please, provide information on alcohol drinking among household members:

| Household head drinks alcohol | Yes/No |
| --- | --- |
| Wife/wives drinks alcohol | Yes/No |
| Other household member drinks alcohol | Yes/No |

49. What is the frequency of drinking alcohol among household members?

| Household head | Daily… Weekly… Monthly… |
| --- | --- |
| Wife/wives | Daily… Weekly… Monthly… |
| Other household member | Daily… Weekly… Monthly… |

50. Please, provide average amount spent by household members on alcohol in a month:

| Person | Amount | Trend compared to previous month |
| --- | --- | --- |
| Household head |  | Increasing,,….. Decreasing……. Same……. |
| Wife/wives |  | Increasing,,….. Decreasing……. Same……. |
| Other household member |  | Increasing,,….. Decreasing……. Same……. |

51. Please, indicate which of the following currently applies to your household:

| Lifestyle | Yes/No | Lifestyle | Yes/No |
| --- | --- | --- | --- |
| Eat more salt |  | Eat more vegetables |  |
| Conscious of the type of vegetable oil used for cooking. |  | Take a lot of soda drinks |  |
| Drink more water |  | Eat less oil |  |
| Take more naps |  | Reduce smoking |  |
| Stop smoking |  | Reduce alcohol |  |
| Stop alcohol |  | Reduce stress |  |
| Increase physical exercises |  | Takes more ginger |  |
| Takes more garlics |  | Takes more moringa |  |
| Take food supplements |  | Reduce hard labour time |  |

52. Do activities involved in cocoa farming lead to any form of body pains? i. Yes … No ……

53 If yes what form of activity? i. Cocoa spraying………….. ii. Cocoa harvesting………. iii. Cocoa farm clearing ………. iv. Establishment of new cocoa farms ……….. v. Others………………..

54. What form of pain do you normally experience? i. Lower back pain ………………… ii. i. Upper back pain iii. General body pain………. iv. Chest pain ………………… v. Others (specify)……..

55. Can you trace the pain to any specific activity on the farm? i. Yes ……………. No ……………

56. If yes, what is the activity? i. Long sitting ……………………. ii. Lifting of heavy objects……… iii. Long distance trekking ……… iv. Long time standing …………. v. Spraying of chemicals ………..

57. How do you treat the pains? i. Self medication …………… ii. Prescriptions from chemists ……… iii. Private clinic …… v. Public hospital ………… v. Traditional doctor ………. vi. Others ………

58. Did any household member secure injuries on farm during this cropping season? i. Yes … No……

59. If yes, provide details below.

| Nature of injury | No. affected | Days incapacitated | Treated by who? | Amount Spent |
| --- | --- | --- | --- | --- |
| Deep cut from cutlasses |  |  |  |  |
| Snake or insect bite |  |  |  |  |
| Falling from a distance |  |  |  |  |
| Chemical poisoning |  |  |  |  |
| Falling down |  |  |  |  |

**Thank you for your precious time to fill this questionnaire.**
